# CGD: a multi-omics database for *Chrysanthemum* genomic and biological research

**DOI:** 10.1093/hr/uhae238

**Published:** 2024-08-21

**Authors:** Jingxuan Ye, Chun Wang, Ye Liu, Shaocong Chen, Jinyu Jin, Lingling Zhang, Peixue Liu, Jing Tang, Jing Zhang, Zhenxing Wang, Jiafu Jiang, Sumei Chen, Fadi Chen, Aiping Song

**Affiliations:** State Key Laboratory of Crop Genetics and Germplasm Enhancement and Utilization, Sanya Institute of Nanjing Agricultural University, Key Laboratory of Landscaping, Ministry of Agriculture and Rural Affairs, Key Laboratory of Biology of Ornamental Plants in East China, National Forestry and Grassland Administration. College of Horticulture, Nanjing Agricultural University, Nanjing 210095, China; Zhongshan Biological Breeding Laboratory, No.50 Zhongling Street, Nanjing 210014, China; State Key Laboratory of Crop Genetics and Germplasm Enhancement and Utilization, Sanya Institute of Nanjing Agricultural University, Key Laboratory of Landscaping, Ministry of Agriculture and Rural Affairs, Key Laboratory of Biology of Ornamental Plants in East China, National Forestry and Grassland Administration. College of Horticulture, Nanjing Agricultural University, Nanjing 210095, China; Zhongshan Biological Breeding Laboratory, No.50 Zhongling Street, Nanjing 210014, China; State Key Laboratory of Crop Genetics and Germplasm Enhancement and Utilization, Sanya Institute of Nanjing Agricultural University, Key Laboratory of Landscaping, Ministry of Agriculture and Rural Affairs, Key Laboratory of Biology of Ornamental Plants in East China, National Forestry and Grassland Administration. College of Horticulture, Nanjing Agricultural University, Nanjing 210095, China; Zhongshan Biological Breeding Laboratory, No.50 Zhongling Street, Nanjing 210014, China; State Key Laboratory of Crop Genetics and Germplasm Enhancement and Utilization, Sanya Institute of Nanjing Agricultural University, Key Laboratory of Landscaping, Ministry of Agriculture and Rural Affairs, Key Laboratory of Biology of Ornamental Plants in East China, National Forestry and Grassland Administration. College of Horticulture, Nanjing Agricultural University, Nanjing 210095, China; Zhongshan Biological Breeding Laboratory, No.50 Zhongling Street, Nanjing 210014, China; State Key Laboratory of Crop Genetics and Germplasm Enhancement and Utilization, Sanya Institute of Nanjing Agricultural University, Key Laboratory of Landscaping, Ministry of Agriculture and Rural Affairs, Key Laboratory of Biology of Ornamental Plants in East China, National Forestry and Grassland Administration. College of Horticulture, Nanjing Agricultural University, Nanjing 210095, China; Zhongshan Biological Breeding Laboratory, No.50 Zhongling Street, Nanjing 210014, China; State Key Laboratory of Crop Genetics and Germplasm Enhancement and Utilization, Sanya Institute of Nanjing Agricultural University, Key Laboratory of Landscaping, Ministry of Agriculture and Rural Affairs, Key Laboratory of Biology of Ornamental Plants in East China, National Forestry and Grassland Administration. College of Horticulture, Nanjing Agricultural University, Nanjing 210095, China; Zhongshan Biological Breeding Laboratory, No.50 Zhongling Street, Nanjing 210014, China; State Key Laboratory of Crop Genetics and Germplasm Enhancement and Utilization, Sanya Institute of Nanjing Agricultural University, Key Laboratory of Landscaping, Ministry of Agriculture and Rural Affairs, Key Laboratory of Biology of Ornamental Plants in East China, National Forestry and Grassland Administration. College of Horticulture, Nanjing Agricultural University, Nanjing 210095, China; Zhongshan Biological Breeding Laboratory, No.50 Zhongling Street, Nanjing 210014, China; State Key Laboratory of Crop Genetics and Germplasm Enhancement and Utilization, Sanya Institute of Nanjing Agricultural University, Key Laboratory of Landscaping, Ministry of Agriculture and Rural Affairs, Key Laboratory of Biology of Ornamental Plants in East China, National Forestry and Grassland Administration. College of Horticulture, Nanjing Agricultural University, Nanjing 210095, China; Zhongshan Biological Breeding Laboratory, No.50 Zhongling Street, Nanjing 210014, China; State Key Laboratory of Crop Genetics and Germplasm Enhancement and Utilization, Sanya Institute of Nanjing Agricultural University, Key Laboratory of Landscaping, Ministry of Agriculture and Rural Affairs, Key Laboratory of Biology of Ornamental Plants in East China, National Forestry and Grassland Administration. College of Horticulture, Nanjing Agricultural University, Nanjing 210095, China; Zhongshan Biological Breeding Laboratory, No.50 Zhongling Street, Nanjing 210014, China; State Key Laboratory of Crop Genetics and Germplasm Enhancement and Utilization, Sanya Institute of Nanjing Agricultural University, Key Laboratory of Landscaping, Ministry of Agriculture and Rural Affairs, Key Laboratory of Biology of Ornamental Plants in East China, National Forestry and Grassland Administration. College of Horticulture, Nanjing Agricultural University, Nanjing 210095, China; Zhongshan Biological Breeding Laboratory, No.50 Zhongling Street, Nanjing 210014, China; State Key Laboratory of Crop Genetics and Germplasm Enhancement and Utilization, Sanya Institute of Nanjing Agricultural University, Key Laboratory of Landscaping, Ministry of Agriculture and Rural Affairs, Key Laboratory of Biology of Ornamental Plants in East China, National Forestry and Grassland Administration. College of Horticulture, Nanjing Agricultural University, Nanjing 210095, China; Zhongshan Biological Breeding Laboratory, No.50 Zhongling Street, Nanjing 210014, China; State Key Laboratory of Crop Genetics and Germplasm Enhancement and Utilization, Sanya Institute of Nanjing Agricultural University, Key Laboratory of Landscaping, Ministry of Agriculture and Rural Affairs, Key Laboratory of Biology of Ornamental Plants in East China, National Forestry and Grassland Administration. College of Horticulture, Nanjing Agricultural University, Nanjing 210095, China; Zhongshan Biological Breeding Laboratory, No.50 Zhongling Street, Nanjing 210014, China; State Key Laboratory of Crop Genetics and Germplasm Enhancement and Utilization, Sanya Institute of Nanjing Agricultural University, Key Laboratory of Landscaping, Ministry of Agriculture and Rural Affairs, Key Laboratory of Biology of Ornamental Plants in East China, National Forestry and Grassland Administration. College of Horticulture, Nanjing Agricultural University, Nanjing 210095, China; Zhongshan Biological Breeding Laboratory, No.50 Zhongling Street, Nanjing 210014, China; State Key Laboratory of Crop Genetics and Germplasm Enhancement and Utilization, Sanya Institute of Nanjing Agricultural University, Key Laboratory of Landscaping, Ministry of Agriculture and Rural Affairs, Key Laboratory of Biology of Ornamental Plants in East China, National Forestry and Grassland Administration. College of Horticulture, Nanjing Agricultural University, Nanjing 210095, China; Zhongshan Biological Breeding Laboratory, No.50 Zhongling Street, Nanjing 210014, China

## Abstract

Asteraceae is the largest family of dicotyledons and includes *Chrysanthemum* and *Helianthus*, two important genera of ornamental plants. The genus *Chrysanthemum* consists of more than 30 species and contains many economically important ornamental, medicinal, and industrial plants. To more effectively promote *Chrysanthemum* research, we constructed the CGD, a *Chrysanthemum* genome database containing a large amount of data and useful tools. The CGD hosts well-assembled reference genome data for six *Chrysanthemum* species. These genomic data were fully annotated by comparison with various protein and domain data. Transcriptome data for nine different tissues, five flower developmental stages, and five treatments were subsequently added to the CGD. A fully functional ‘RNA data’ module was designed to provide complete and visual expression profile data. In addition, the CGD also provides many of the latest bioinformatics analysis tools, such as the efficient sgRNA search tool for *Chrysanthemum*. In conclusion, the CGD provides the latest, richest, and most complete multi-omics resources and powerful tools for *Chrysanthemum*. Collectively, the CGD will become the central gateway for *Chrysanthemum* genomics and genetic breeding research and will aid in the study of polyploid evolution.

## Introduction

As the largest dicotyledonous family, Asteraceae (also called Compositae) consists of more than 25 000 species in approximately 1600 genera and is widely distributed worldwide [1]. The family includes many important genera of industrial and ornamental plants, such as *Chrysanthemum* and *Helianthus*. Genus *Chrysanthemum*, as an important part of the Compositae family, contains many plants with the ornamental, medicinal and industrial value. *Chrysanthemum morifolium*, a perennial herbaceous plant that originated in China, has a history of cultivation comprising more than 3000 years [2, 3]. Since the eighth century Ad, Japan and Europe have introduced cultivated chrysanthemum cultivars from China and bred many varieties [4]. At present, chrysanthemums have become one of the most economically important flowers in the world [5]. In October 2018, the genome of the first wild diploid species of the genus *Chrysanthemum*, *Chrysanthemum nankingense*, was completely sequenced and resolved [6]. In the following 6 years, the genomes of the diploid wild *Chrysanthemum* species *Chrysanthemum seticuspe* [7], *Chrysanthemum lavandulifolium* [8], *Chrysanthemum makinoi* [9], and *Chrysanthemum indicum* [10] were obtained and reported. In April 2023, the first genome of cultivated chrysanthemum (*C. morifolium* Ramat., Chinese name ‘Ju Hua’) was resolved and formally published [11]. These valuable data have laid a solid foundation for basic research and industrial development of genus *Chrysanthemum*.

In recent years, the development of multi-omics technologies has provided new opportunities for researchers to analyse crop growth and development from multiple dimensions [12, 13]. Based on these multi-omics data, various multi-omics analysis tools, such as genome-wide association studies (GWASs), BSA-seq and weighted correlation network analysis (WGCNA) have been developed and widely used in genetic analysis and breeding. These techniques have demonstrated the power of multi-omics in assisted breeding [14–16]. To better utilize multi-omics data, multi-omics platforms have been developed for numerous important crops, such as Brassicaceae [17], Cucurbitaceae [18], the genus *Camellia* [19], rice (*Oryza sativa* L.) [20], wheat (*Triticum aestivum* L.) [21], maize (*Zea mays* L.) [22], tomato (*Solanum lycopersicum*) [23], and rapeseed (*Brassica napus*) [24]. The lack of a multi-omics platform that integrates multi-omics data and bioinformatics tools has greatly slowed the progress of basic research and genetic breeding in chrysanthemum.

To this end, we constructed the *Chrysanthemum* genome database (CGD) (CGD, http://210.22.121.250:8880/asteraceae/homePage), the first *Chrysanthemum* bioinformation center. The CGD contains all the most recently published genomic data, rich transcriptome data, and newly developed or integrated bioinformatics tools. We believe that the use of multi-omics data and tools integrated in the CGD can accelerate basic research on *Chrysanthemum* and provide effective tools for assisted genetic breeding, thus promoting germplasm innovation in chrysanthemum and flourishing development in the chrysanthemum industry.

## Database contents

### *Chrysanthemum* assemblies and syntenies

The results and data of previous sequencing efforts for a total of six *Chrysanthemum* species, including one cultivated chrysanthemum (*C. morifolium*, ‘Zhongshanzigui’), two edible chrysanthemum (*C. nankingense* and *C. indicum*) and three wild *Chrysanthemum* species (*C. seticuspe*, *C. makinoi*, and *C. lavandulifolium*), have been published in the CGD (Table 1). In addition, genomic synteny blocks and syntenic gene pairs were identified between any two *Chrysanthemum* genome assemblies and within each of the other five *Chrysanthemum* genome assemblies. The protein sequences of the six *Chrysanthemum* genome assemblies were aligned with each other by BLASTP [25], with an E value of 1e^−10^ and a maximum of five alignments. The alignment results were subsequently input to MC-ScanX [26] to identify genomic blocks and syntenic gene pairs using default parameters. In total, 55 376 genomic syntenic blocks containing 1261 865 gene pairs were identified between or within six *Chrysanthemum* genome assemblies based on the results of the collinearity analysis (Table S1, see online supplementary material).

**Table 1:** Information on the six *Chrysanthemum* genome assemblies

**Latin name**	**Accession**	**Version**	**No. genes**	**source**
*Chrysanthemum indicum*		v1	50 606	[10]
*Chrysanthemum lavandulifolium*	G_1_	v1	64 257	[8]
*Chrysanthemum makinoi*	JP131333	v1	95 064	[9]
*Chrysanthemum morifolium*	Zhongshanzigui	v1	138 749	[11]
*Chrysanthemum nankingense*		haplotype	43 821	[11]
		diplotype	74 035	
*Chrysanthemum seticuspe*	Gojo-0	v1	74 259	[7]

**Table 2:** Gene functional annotations of six *Chrysanthemum* genomes

**Species**	**NR**	**SwissProt**	**GO**	**KEGG**	**Interpro**
*Chrysanthemum indium*	47 727	33 252	24 452	13 330	50 606
*Chrysanthemum lavandulifolium*	60 095	36 466	25 547	19 244	603
*Chrysanthemum makinoi*	77 601	44 239	34 547	19 708	657
*Chrysanthemum morifolium*	131 800	94 067	63 844	48 693	1605
*Chrysanthemum nankingense* (Cn_A)	42 690	31 521	21 505	17 575	547
*Chrysanthemum nankingense* (Cn_AB)	72 156	53 436	36 434	29 762	914
*Chrysanthemum seticuspe*	69 493	44 690	23 792	25 480	647

**Figure 1:**
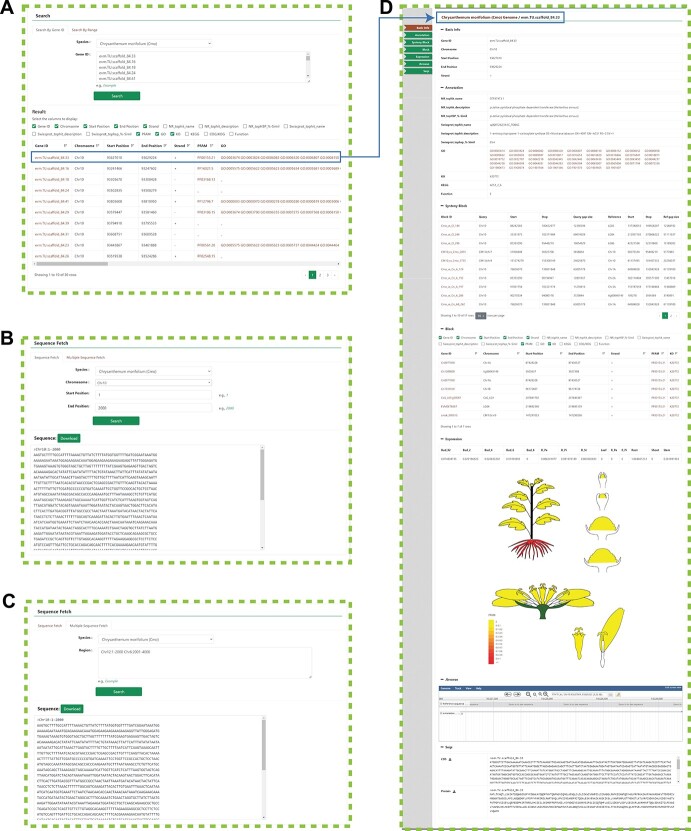
Gene interface and search function in the CGD. **A** Gene ID search and results page. **B** and **C** Sequence fetch and results page. **D** Gene details page, with seven function module navigation bars on the left and an overview of all the gene information on the right.

**Figure 2:**
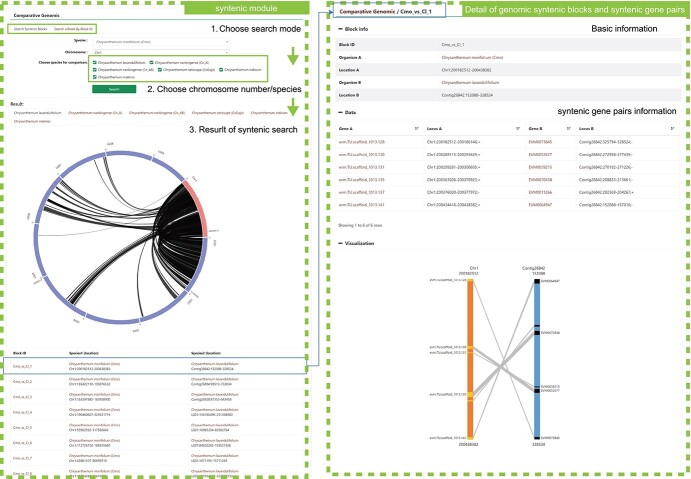
Synteny analysis of six *Chrysanthemum* species.

**Figure 3:**
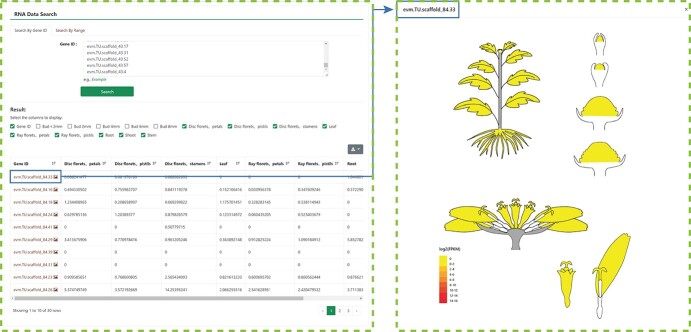
Expression module in the CGD. **A** Search by gene ID in the RNA Data module. **B** Search by range page in the RNA Data module. **C** visual expression profile page in the RNA Data module.

**Figure 4:**
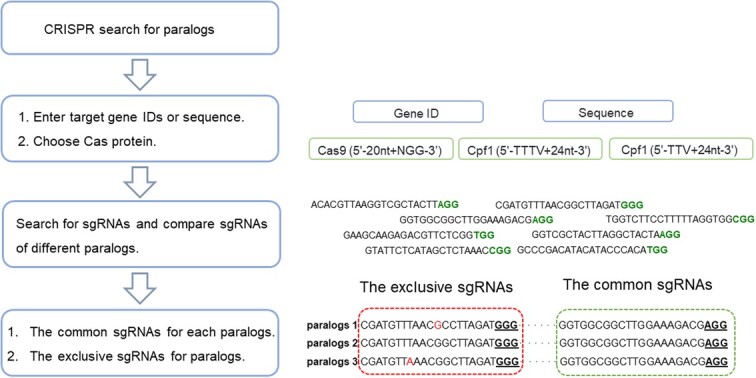
Workflow of the CRISPR search for paralogs.

### *Chrysanthemum* genes and annotations

A total of 540 791 protein-coding genes predicted in six *Chrysanthemum* species were comprehensively annotated using various public protein and domain databases. The protein sequences of the protein-coding genes were compared against the NCBI nonredundant protein database (NR) [27] and SwissProt protein database [28] using BLASTP with default parameters. A total of 501 562 and 337 671 *Chrysanthemum* genes were found to be homologous genes in the NCBI NR and SwissProt databases, respectively. EggNOG-Mapper, KofamScan, and interProsScan were used to annotate the Gene Ontology (GO) [29], KEGG [30], and interPro [31] categories of *Chrysanthemum* genes with default parameters. A total of 55 579, 230 121, and 173 792 genes annotated to the interPro, GO, and KEGG categories, respectively (Table 2). All the annotations of the *Chrysanthemum* species are stored and well organized in the CGD.

### *Chrysanthemum* transcriptomes

The raw RNA-seq data in the CGD is downloaded from the NCBI Sequence Read Archive (SRA). In order to better manage the data, CGD provides a concise description of the transcriptome data by collating summary information or publications of the source data. RNA-seq data lacking clear sampling or treatment information will not be included in the CGD. After removing low-quality read segments, rRNA, and splitter sequences, the final clean reads are aligned to the reference genome. Following alignment, the original count of each protein-coding gene is calculated and then normalized to fragments per kilobase per million mapped fragments (FPKM). Currently, the *C. morifolium* gene expression profiles across nine organs (roots, stems, shoot, leaves, and flower organs dissected from ray florets and disc florets), five flower bud developmental stages (<2 mm, 2 mm, 4 mm, 6 mm, and 8 mm), three exogenous substance treatments (melatonin treatment, ethephon treatment, and cold treatment) and two biotic stress treatment (*Alternaria alternata* inoculation and *Puccinia horiana* inoculation) are also available in the CGD.

## Database functions

### Gene interface module

Based on our genome sequencing and gene annotation work, the *Chrysanthemum* species gene retrieval system was established. The CGD provides basic gene search functions, such as searching by gene number and batch search functions (Fig. 1A–C). To provide users with a quick and comprehensive understanding of gene information, the CGD provides seven sections on the gene detail page: ‘Basic info’, ‘Annotation’, ‘Synteny Block’, ‘Block’, ‘Expression’, ‘Jbrowse’, and ‘Seqs’ (Fig. 1D). The ‘Basic info’, ‘Annotation’, ‘Jbrowse’, and ‘Seqs’ modules provide basic information about genes, annotations, chromosome location, and sequence information. In addition to providing basic gene information, ‘Synteny Block’ and ‘Block’ also provide orthologous and paralogous genes of interest in different synteny blocks, and users can intuitively obtain all gene location information by visualizing genomic synteny block data (Fig. 2). ‘Expression’ contains RNA expression profile information from different tissues and treatments, allowing the user to quickly acquire expression profiles of target genes. All in all, the user can easily access any gene-related information in the ‘Gene interface’

### RNA data module

To better display the expression profile data, the CGD homepage provides an ‘RNA data’ module where users can obtain expression profiles by choosing either to search by gene ID (Fig. 3A) or to search by range (Fig. 3B). In addition, the expression profiles of the genes of interest in different RNA-seq items can be accessed through the ‘Expression’ module provided on the gene page. Furthermore, the eFP browser module was added to the CGD for visualization of all the expression profiles (Fig. 3C). Now, the expression profile data of specific genes can be accessed simply and directly.

### CRISPR module

Gene editing technology plays a key role in realizing precision-directed breeding in crops. CRISPR (clustered regularly interspaced short palindromic repeats)-Cas (CRISPR associated) has evolved into one of the most advanced crop genetic engineering systems and has been widely used in ornamental crops [32, 33]. To better support the development of gene editing for *Chrysanthemum* breeding, we developed the *Chrysanthemum* CRISPR search system by integrating all the publicly released genomic data of genus *Chrysanthemum*, CRISPR-P v2.0, and CRISPR-local system [34, 35]. This function includes three Cas protein guide sequence design strategies: SpCas9, LbCas12a, and FnCas12a, which provides a variety of toolkits for *Chrysanthemum* gene editing. In ‘CRISPR search’ module, users can obtain the sgRNA sequence of the target gene by inputting one gene number of the target gene and selecting the sgRNA according to the score and the probability of off-target editing events given by the system. In addition, we optimized and developed a CRISPR search module for editing multiple paralogs for complex polyploid characteristics of cultivated chrysanthemums, which is called CRISPR search for paralogs (Fig. 4). Now, users can use this system to design generic targets for paralogs or specific targets for individual genes within paralogs.

### Other tools

In addition to the above bioinformatics tools, many useful genome analysis tools, such as Muscle, GeneWise, GO, and KEGG enrichment, and transcriptome analysis tools, such as correlation heatmaps and co-expression networks, have been added to the CGD. To achieve a great representation of the large amount of multi-omics data, we implemented a variety of data visualization methods in addition to expression data. In the comparative genomics module, genomic syntenic blocks and syntenic gene pairs between uniform and different species can be easily obtained. These common and useful bioinformatics analysis tools embedded in databases can help users analyse *Chrysanthemum* multi-omics data. In the future, we will add more useful bioinformatics tools to the CGD and continue to optimize the suitability of existing tools in the CGD.

## Discussion and perspectives

In this study, we established the CGD, the first *Chrysanthemum* database based on genomic and transcriptome data. The CGD provides the most up-to-date multi-omics dataset of *Chrysanthemum* species to date, including genomic information, gene sequences and annotations, and spatiotemporal information on gene expression. Based on these complete and rich data, we have developed and integrated numerous useful bioinformatics analysis and visualization tools. This approach will help researchers understand the evolutionary relationships of different *Chrysanthemum* species, clarify the regulatory networks of growth and development, and uncover key genes involved.

In conclusion, these data and tools fully demonstrate the powerful potential of the CGD in basic research and genetic breeding. In the future, we will strengthen our collaboration with *Chrysanthemum* researchers around the world. In addition, we will regularly collect new datasets to analyse and add to the CGD, including but not limited to genomes (for *de novo* assembly and resequencing), proteomes, transcriptomes (for single-cell transcriptomes and spatiotemporal transcriptomes), epigenomes, metabolomes, phenomes, and phenotypes. We believe that the CGD will become a central gateway for scientific research on *Chrysanthemum* species in the near future, further extending its potential and thus promoting the development of the chrysanthemum industry.

## Supplementary Material

Web_Material_uhae238

## Data Availability

All the data hosted in the CGD are freely available at (http://210.22.121.250:8880/asteraceae/homePage).
